# Endothelial Nitric Oxide Synthase (eNOS) Gene Polymorphism is Associated with Age Onset of Menarche in Sickle Cell Disease Females of India

**DOI:** 10.4084/MJHID.2013.036

**Published:** 2013-06-04

**Authors:** Sudhansu Sekhar Nishank

**Affiliations:** Regional Medical Research Centre for Tribals (ICMR), Nagpur Road, PO-Garha, Jabalpur-482003, Madhya Pradesh, INDIA

## Abstract

**Background and Objective:**

Females with sickle cell disease (SCD) often show late onset of menarche. In transgenic sickle cell mouse, deficiency of gene encoding endothelial nitric oxide synthase (*eNOS*) has been reported to be associated with late onset of menarche. Thus to explore the possible association of *eNOS* gene polymorphism with age of onset of menarche in SCD females, 3 important *eNOS* gene polymorphisms- *eNOS 4a/b*, *eNOS 894G>T* (rs1799983) and *eNOS-786 T>C* (rs2070744) and plasma nitrite levels were tested among three groups of females- SCD late menarche, SCD early menarche and control females.

**Methodology:**

About 39 SCD females comprising of 18 SCD early menarche and 21 SCD late menarche groups were studied along with 48 control females. Genotyping of eNOS gene polymorphisms were done by PCR-RFLP and quantification of plasma nitrite level was performed by ELISA based commercial kits.

**Results:**

SCD late menarche females showed significantly higher prevalence and higher association of heterozygous genotypes, higher frequency of mutant alleles *.4a., .T.* and *.C.* as compared to that of control group and SCD early menarche group. The frequency of haplotype .*4a-G-C*. and haplotype .*4b-G-C*. (alleles in order of *eNOS 4a/b*, *eNOS 894G>T* and *eNOS-786 T>C* respectively) were found to be significantly high in SCD late menarche compared to combined groups of SCD early menarche and controls. SCD late menarche group had significantly low level of plasma nitrite concentration for all 3 *eNOS* gene polymorphisms as compared to controls and SCD early menarche females.

**Conclusion:**

*eNOS* gene polymorphism may influence age of onset of menarche in SCD females.

## Introduction

Menarche is the first menstrual cycle often considered as the central event of female puberty in humans. Onset of menarche is found to be associated with the endometrium derived nitric oxide (NO).^1^ Nitric oxide is a major paracrine mediator and important regulatory agent in various female reproductive processes such as menstruation, ovulation, implantation, pregnancy maintenance, labor and delivery. Circulatory NO products increase during follicle development and decline immediately after ovulation.^1^,^2^ In primates, NO has been found to be involved in the initiation and maintenance of menstrual bleeding by inducing tissue breakdown and vascular relaxation as well as by inhibiting platelet aggregation. Endometrium derived NO also plays an important role in myeometrial relaxation during menstruation.^3^ NO is synthesized by 3 isoforms of nitric oxide synthase (NOS) enzyme, such as endothelial NOS (*eNOS* or *NOS III*), inducible NOS (*iNOS* or *NOS II*) and neuronal NOS (*nNOS* or *NOS I*). NOS is encoded by the gene located on the long arm of chromosome 7 (7q35–36). Immunohistochemical and molecular studies of endometrial NOS expression *in vivo* with NOS inhibitors indicate that *eNOS* mRNA is expressed throughout menstrual cycle of human.^4^ Throughout the course of menstrual cycle endometrium derived NO plays an important role as signaling molecule to bring about various functions such as intense vasoconstriction, onset of bleeding and subsequent hemostasis in human females as well as other mammalian females.^5^ Nitric oxide produced in human endometrium plays a central role in the control of menstruation (and implantation) as a vasodilator and an inhibitor of platelet aggregation.^5^ The level of NO in the body is linked to expression of different polymorphic variants of *eNOS* gene, the most important being *eNOS 894G>T* (rs1799983) in exon 7, *eNOS 4a/b* ( a 27bp VNTR repeat in intron 4) and *eNOS -786 T>C* (rs2070744) in 5′ promoter region. Mutation in these important polymorphic variants leads to decreased production of nitric oxide in humans.^6^ In mouse model, *eNOS* (*NOS3*) deficiency has been reported to be associated with late onset of menarche.^7^ Study conducted on healthy Caucasian women in Austria did not find any association between polymorphic variants of *eNOS* gene ( uch as *894G>T, - 786T>C*) and age onset of menarche.^8^

Majority of reports on association of eNOS and menarche are based on studies on normal population. However, these reports are lacking on SCD women. SCD patients show reduced bioavailability of NO as well as late onset of menarche.^9^ An earlier study in state of Odisha (India) found late onset of menarche among SCD females (14.9 ± 1.55 years) compared to that of normal females (13.7 ± 1.37 years).^10^ Moreover, reports are lacking on the role of genetic factors linking menarche and eNOS gene polymorphism with the age of onset of menarche in SCD females. Therefore the present study was carried out to identify possible association of eNOS gene polymorphisms with the age of onset of menarche among SCD females.

## Materials and Methods

### Study Subjects

SCD females attending sickle cell clinic [of Regional Medical Research Centre for Tribals (ICMR) situated in the campus of NSB Medical College, Jabalpur, Madhya Pradesh] for regular follow up were the study population. Age of menarche (age at first menstrual period in years) was documented based on published data. In India ideal age of menarche in pubescent girls ranges from 12 to 14 years with mean of 13.71 (±1.37) year.^10^,^11^ Those girls showing age of onset of menarche before this published ideal age range was designated as early menarche girls whereas those having age onset of menarche later than this published ideal range were designated as late menarche group. Similarly body height and weight were measured and arranged into late menarche and early menarche group. Age matched and ethnicity matched normal females (having normal hemoglobin phenotype) referred to the department of Genetics for routine diagnosis for haemoglobinopathies were used as control for comparison of *eNOS* gene polymorphism. All study subjects belonged to Scheduled Caste, scheduled Tribe, Other Backward Caste and General communities of state of Madhya Pradesh only. Peripheral blood sample from 39 SCD females and 48 normal/control belonging to different families were collected between March 2012 to October 2012. Among 39 SCD females, 21 females reported late onset of menarche (SCD late menarche) and 18 females reported early onset of menarche. Blood sample for DNA analysis and plasma samples were collected after written consent from the patient and/or her parents/legal guardians. The study was approved by ethical committee of RMRCT (ICMR), Govt. of India and NSB Medical College, Govt. of Madhya Pradesh. Besides the present study was conducted in accordance with ethical standards of Helsinki Declaration.

### DNA Isolation and Genotyping

Human genomic DNA was extracted according to manufacturer’s instruction using blood genomic DNA extraction kit (Fermentas, Germany). The T→C transition at position -786 in the 5′ flanking region of the *eNOS* gene was determined by performing PCR-restriction fragment length polymorphism analysis using forward primer 5′-GAGTCTGGCCAACACAAATCC-3′ and reverse primer 5′-GACCTCTAGGGTCATGCAGGT-3′. The PCR fragment (657bp) was digested with *Hpa II* restriction enzyme by overnight incubation at 37°C as reported earlier.^12^ The wild type sequence (*-786T*) was not cleaved whereas the mutant sequence (*-786C*) was cleaved into 2 fragments (373bp and 284bp). Homozygous mutants (CC) produced 2 PCR fragments-327 bp, 284 bp; heterozygous mutant (TC) produced 3 fragments- 373 bp, 327 bp, and 284 bp; whereas homozygous wild (TT) produced 2 fragments-373 bp and 284 bp. Genotyping of the *eNOS 894G>T* polymorphism was done by PCR amplification of exon 7 using sense primer 5′-AAG GCA GGA GAC AGT GGA TGGA-3′ and antisense primer 5′ – CCCAGT CAA TCC CTTT TGG TGC TCA-3′ followed by digestion of PCR product (248bp) with *Mbo I* restriction enzyme by overnight incubation at 37°C as described earlier^12^ . The mutant allele *894T* was cleaved into 2 fragments (158 and 90bp). The 27bp repeat VNTR in intron 4 (*eNOS 4a/b*) was differentiated only by allele specific PCR. The sequences of flanking primers used was 5′-AGG CCCTAT GGT AGT GCC TTT-3′ (sense) and 5′-TCT CTT AGT GCT GTG GTC AC-3′ (antisense). Mutant allele *eNOS 4a* (4 repeats) produced 393bp fragment and wild type allele *eNOS 4b* (5 repeats) produced 420bp fragment as reported earlier.^14^ The PCR condition for each of these SNP of eNOS gene was: a 25μl reaction volume containing 100ng of template genomic DNA, 10pmol of each primer, 200μM of each dNTP, 1.5mmol/L MgCl^2^, 2.5μl of 10xPCR buffer and 2U of DNA Taq Polymerase (Fermentas, Germany). The PCR mixture was heated to 94°C for 5min for denaturation and kept for 35 cycles each of denaturation at 94°C for 30sec, annealing at 60°C ( but 65.5°C for *eNOS -786 T>C* SNP) for 40sec, extension at 72°C for 1 min followed by final extension at 72°C for 5 min. The PCR product was run in 2.5% agarose gel and gel image was captured by Gel Doc. To ensure that there was no error in genotyping, about 10% of the randomly selected samples were regenotyped for intron *4VNTR* (*eNOS 4ab*) and *- 786T>C*, whereas genotyping for the *894G>T* was repeated for all of the samples and the results were found to be 100% concordant.

### Plasma Nitrite (NO_2_) Assay

Plasma samples from patients with acute renal failure and patients under medications, such as long acting nitrates (sorbitate), were excluded from the study to avoid interference with plasma nitrite measurement. Plasma from patients who had consumed low nitrite food 12 hour prior to blood draw were included. In order to eliminate the possibility of nitrate contamination of EDTA tubes, the tubes were prewashed with Milli Q (Millipore, USA) water. In addition, the EDTA solution used had undetectable levels of nitrite. Plasma nitrite (NO_2_) was measured after enzymatic conversion of NO_3_^−^ to NO_2_^−^ by nitrate reductase in duplicates according to the manufacturer’s instructions using a commercial ELISA based kit (Enzo life Science, Switzerland).

### Statistical Analysis

All statistical analysis were performed with statistical software Graph Pad Prism (version 5.0, USA). The association of genotypes and allele frequencies between the two clinical groups was determined by computing the odds ratio derived from a Fisher exact test two tailed. The *X*^2^ test was also used to test the Hardy-Weinberg equilibrium. Statistical significance was defined as a *P* value of < 0.05. Linkage disequilibrium was examined by *X*^2^ analysis, and the extent of disequilibrium was determined as follows D′ = D/D_max_. The SNP Alyze program (version 8.0, Dynacom Corporation, Japan) based on the expectation maximization algorithm, was used to estimate the maximum likelihood of haplotype frequencies in each group and to identify which specific haplotypes were associated with a clinical outcome of SCD patients. *P* value of < 0.00625 (0.05/ number of haplotypes) was considered significant to correct for the number of comparisons made. The median plasma nitrite levels were compared by using Mann-Whitney U test.

## Result

**Figure 1:**
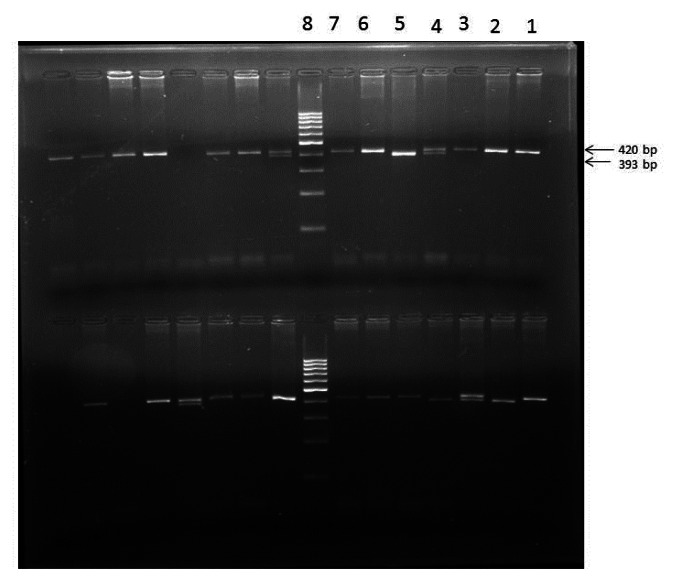
eNOS 4a/b polymorphism showing homozygous wild (bb) in lanes 1,2,3,6,7 (of upper row); heterozygous mutant (ab) in lane 4; homozygous mutant (aa) in lane 5.

**Figure 2:**
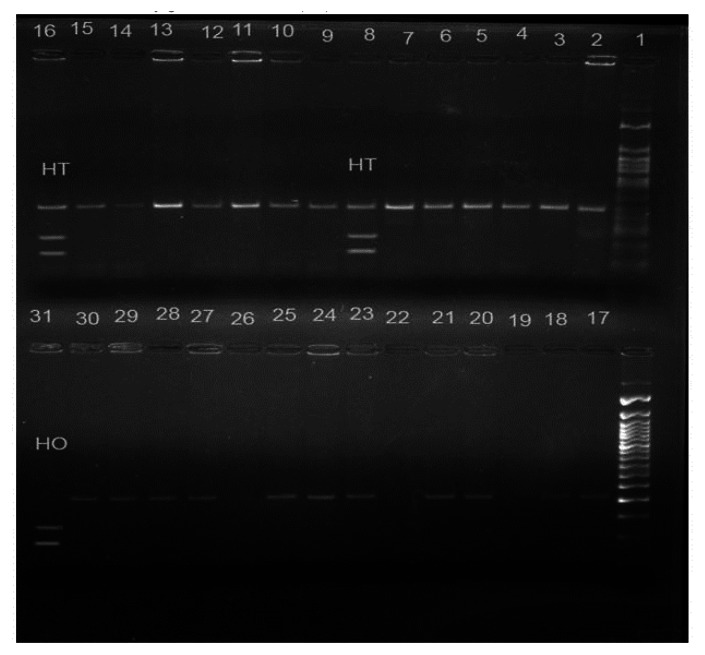
eNOS 894 G>T polymorphism showing heterozygous mutant (GT) in lanes 8 and 16; homozygous mutant (TT) in lane 31; 50 bp molecular marker in lane 1; homozygous wild (GG) in rest of the lanes.

**Figure 3:**
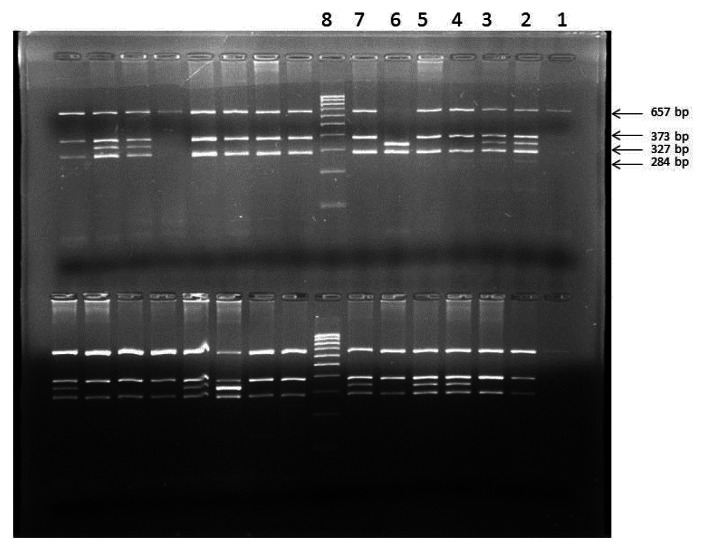
eNOS -786 T>C polymorphism showing in upper row (from right to left) PCR undigested product in lane 1; heterozygous mutant (TC) in lanes 2, 3; homozygous wild (TT) in lanes 4,5 and 7, homozygous mutant (CC) in lane 6, Molecular marker (100 bp) in lane 8.

The mean age onset of menarche in the present study was found to be 13.1 years (±1.4, Standard Deviation) for control group, 12.6 years (±0.8, SD) for SCD patients with early onset of menarche (SCD early menarche) and 17.1 years (±1.65, SD) for SCD patients with late onset of menarche (SCD late menarche). The median age along with interquartile age range of menarche for these groups were 13.0 (12.0 – 14.0) years in controls; 12.3 (12.0 – 13.25) years in SCD early menarche and 16.5 (15.6 – 18.5) years in SCD late menarche groups. The mean height and mean body weight of girls belonging to early menarche group were found to be 154.1(±2.24) cm and 45.76 (±3.27) Kg respectively which were significantly higher (*P*< 0.05) than that of late menarche group (Height: 150.5 ± 2.47 cm and Weight 43.05 ±1.23 kg.) (Table not shown). The photographs of different *eNOS* gene polymorphisms are shown in Figure 1 (*eNOS 4ab*), Figure 2 (*eNOS894G>T*) and Figure 3 (*eNOS - 786T>C*). The genotype and allele distribution of *eNOS* gene polymorphism particularly *eNOS 4a/b, eNOS 894G>T, eNOS -786T>C* have been summarized in Table 1 and Table 2. As compared to control group, SCD late menarche group (Table 1) had significantly higher prevalence of heterozygous genotypes, *4a/4b* of *eNOS 4a/b*, G/T of *eNOS 894G>T*, T/C of *eNOS - 786T>C* along with higher frequencies of mutant alleles ‘*4a. .T.* and *.C.* of *eNOS* gene polymorphisms. On the other hand control group had significantly higher prevalence and association of homozygous wild type genotypes, *4b/4b, G/G, T/T* as compared to SCD late menarche (*P*< 0.0001). Comparison of genotype and allele frequencies between SCD late menarche and SCD early menarche group (Table 2) revealed higher incidence of homozygous wild type genotypes in SCD early menarche whereas higher incidence of heterozygous genotypes along with higher prevalence of mutant alleles of *eNOS* gene polymorphisms were observed in SCD late menarche group. The association of heterozygous genotypes with SCD late menarche was found to be 27.6 fold for *eNOS4a/b*, 19.1 fold for *eNOS894G>T*, 7.0 fold for *eNOS-786T>C* polymorphisms in contrast to SCD early menarche.

The genotype distribution of 3 SNPs of *eNOS* gene did not deviate from Hardy-Weinberg equilibrium in the control group as well as SCD population. Comparison of overall haplotype distribution profiles (Table 3) revealed statistically significant differences between SCD late menarche and control group combined with SCD early menarche group. The genotype and allelic frequencies also did not show any significant difference between SCD early menarche and control group (data not shown). It was observed that the incidence of haplotype-1 (*4b-G-T*) (alleles in order of *eNOS 4a/b, eNOS 894G>T* and *eNOS-786 T>C* respectively) was found to be highly elevated in control cum SCD early menarche group. There were significant association and higher incidence of haplotype-2 (*4a-G-C*) and haplotype-3 (*4b-G-C*) in SCD late menarche as compared to combined SCD early menarche and control groups (Table 3). When plasma nitrite (NO_2_) concentration was considered according to genotypes (Table 4), it was found that there were significant differences in plasma nitrite concentration in SCD late menarche group for all 3 eNOS gene polymorphisms. However the plasma nitrite level did not show any significant difference between the eNOS genotypes in control as well as SCD early menarche groups. The mean level of plasma nitrite was found to be significantly low 224.5 μM (± 23.9, SD) in SCD late menarche group as compared to SCD early menarche (266.7 μM ± 11.7, SD) and control females (264.8 μM ± 9.8, SD) (*P*<0.0001) (data not shown). Table 5 summarizes the results of *D.* values and ‘*P*’ values for linkage disequilibrium (LD) between 3 SNPs of eNOS gene for all studied samples. A strong and significant linkage disequilibrium between *eNOS 4a/4b* and *eNOS 894G>T* (*D*′= 0.511, *P* = 0.0025) and *eNOS 4a/4b* and *eNOS-786T>C* (D′ = 0.674, *P* = 0.01) but only weak and insignificant association between *eNOS 894G>T* and *eNOS - 786T>C* polymorphisms (*D.*= 0.179, *P* = 0.961) were observed.

## Discussion

The present study shows that *eNOS* gene polymorphism is associated with age of onset of menarche among SCD females in India. Our data indicates that heterozygous carriers of *eNOS* gene polymorphism particularly *eNOS 4a/4b* has 13.9 fold, *eNOS 894G>T G/T* has 11.5 fold, *eNOS -786T>C T/C* has 14 fold higher risk of late onset of menarche in SCD females as compared to control groups. The present findings are contradictory to earlier findings on healthy and normal Caucasian females which showed complete absence of association of menarche with these *eNOS* polymorphisms.^8^ Our study reveals that the mean age of menarche of 13.1 years normal/controls was delayed by 4.0 years in SCD females as observed in Jamaican SCD girls^9^ which indicates that ethnic variation may be associated with menarche.

Our findings of higher body weight and height among early menarche females as compared to late menarche females corresponds to earlier findings of Indian study by Bagga and Kulakarni (2000) on normal females and Jamaican study by Serjeant et al (2000) on SCD females.^9^,^11^ This implies that skeletal maturation is associated with menarcheal age besides *eNOS* gene polymorphism.

The higher incidence of mutant genotypes of *eNOS* polymorphisms along with low level of plasma nitrite in present study among SCD late menarche group reveals that SCD late menarche females produce low level of plasma nitric oxide (NO). Earlier studies on healthy normal population have shown that heterozygotes and mutant homozygotes of 3SNPs of *eNOS* gene viz. *eNOS894 G>T, eNOS -786T>C,* and *eNOS 4a/b* are associated with low plasma nitrite and nitrate concentration.^6^,^15^,^16^,^17^,^18^ It has also been observed that there is a significant increase in NO levels in the mammalian ovary along with increased *eNOS* expression during follicular growth.^19^,^20^ Studies have shown that nitric oxide synthase (NOS) activity and rate of production of nitric oxide undergo variation in close association with follicular development in women undergoing in vitro fertilization.^21^ Further there is significant increase in nitric oxide production in women during the middle part of menstrual cycle where the highest nitric oxide (NO) concentration is accompanied by mid cycle increase in estradiol, estrone, luteinizing and follicle stimulating hormones.^22^ Moreover nitric oxide has been found to regulate follicular fluid accumulation of the preovulatory follicles towards ovulation by controlling capillary vessel permeability and participating in the preovulatory modulation of ovarian fluid flow by its vasodilatory activity in mammals.^21^,^23^,^24^,^25^

These studies indicate that early maturation of follicle accompanied with increased synthesis of nitric oxide may be the cause of early onset of menarche in females. It has been reported by earlier studies that specific haplotypes of eNOS gene such as ‘*4a-T-T.*, ‘*4b-G-C.* and ‘*4b-T-C.* (alleles in order of *eNOS 4a/b, eNOS 894G>T, eNOS -786T>C*) are found to be associated with low level of plasma nitrite concentration in normal population.^26^,^27^,^28^ This is also reflected in the present study where ‘*4b-G-C*’ haplotype (Haplotype-3 in present study) and haplotype 4a-G-C ( Haplotype-2) have been found in higher proportion along with low level of nitric oxide among SCD late menarche group than in control group, whereas haplotype ‘*4b-G-T*’( Haplotype-1) was found in higher proportions besides high level of plasma nitric oxide among controls combined with SCD early menarche group. The higher incidence of haplotype ‘*4a-G-C*’ in SCD late menarche may be due to low production of NO in these patients and also due to strong linkage disequilibrium between *eNOS 4a/b* and *eNOS -786T>C* (*D*′ = 0.674) in the study population. The present report is the first of its kind to show role of *eNOS* polymorphisms in SCD females showing late onset of menarche in Indian population.

The present study is limited by small sample size (both for SCD early menarche and late menarche, non-availability of late menarche females from control/normal group besides its small sample size), absence of records/studies on secondary sexual development, hormonal factors, nutritional factors besides body girth viz. of chest, hip, mid arm and calf etc.. The role of nutritional factors and socioeconomic status may determine age onset of menarche among normal females in India.^11^ But nutritional status may not be important in present study because of the fact that majority (>90%) of study subjects from SCD and control group belonged to below poverty line group as defined by Government of India. A recent study of candidate genes associated with age onset of menarche assigned 42 SNPs of 9 genes (*FSHB, LHCGR, POMC, UGT2B4, GHRH, CD40LG, FGFR1, KISS1, NKX2-1*) among Caucasian normal females.^29^ Thus, the present finding of association between *eNOS* gene and menarche, assign *eNOS* gene to be an additional genetic modulator of menarche in SCD females. In addition to this human study, an earlier report has also revealed association of menarche with eNOS gene in mouse.^14^

Nitric oxide act as an important mediator of uteroplacental blood flow and uterine quiescence during pregnancy. It modulates placental hormone production and influences placental human chorionic gonadotropin production during gestation. Deficient *eNOS* gene and reduced synthesis of nitric oxide, in mice models and human, have been found to be associated with impaired fetal development, placental abruption, recurrent miscarriages and death of fetus in utero.^7^,^30,31^ Thus SCD pubescent girls showing late onset of menarche (because these females are associated with mutant *eNOS gene* and low level of nitric oxide) may be at risk of acquiring these morbidities during pregnancy in future as compared to early menarche group. This is further evidenced by accumulating reports of increased association of morbidities among SCD females during pregnancy throughout the world including India.^32,33,34,35^ However this need large sample based future studies taking several associating genetic and environmental factors.

There is paucity of reports on *eNOS* gene and age onset of menarche in SCD females throughout the world. Although the present study sample size is small and carries limitations, in view of significant incidence of *eNOS* gene polymorphism among late menarche SCD females who are likely to be susceptible to morbidities during pregnancy in future, *eNOS* gene may be thought to be a genetic modifier of menarche in SCD females necessitating further evaluation carrying larger sample size.

## Figures and Tables

**Table 1:** Genotype and allele frequencies for various eNOS polymorphisms in SCD patients with late menarche and normal/control group.

Genotype or alleles	SCD late menarche (n=21)	Control/normal (n=48)	Odds ratio† (95% confidence interval)	*P* value†
eNOS 4a/b				
4b/4b	6 (28.5%)	43(89.5%)	0.046 (0.0123 −0.174 )	< 0.0001
4a/4b	13 (62.0%)	5(10.4%)	13.9 (3.89 – 50.18)	< 0.001
4a/4a	2 (9.5%)	0		NS
Alleles				
4b (wild)	25 (0.595)	91 (0.947)	0.0808 (0.0271 – 0.2406)	<0.0001
4a (mutant)	17 (0.404 )	5 (0.052)		
eNOS 894G>T				
G/G	13 (62.0%)	46 (95.8%)	0.0706 (0.0133 – 0.374)	0.0007
G/T	7 (33.3%)	2 (4.16%)	11.5 ( 2.139 – 61.82)	0.0025
T/T	1 (4.7%)	0		NS
Alleles				
G (wild)	33 (0.785)	94 (0.979)	0.078 (0.016 – 0.379)	0.0004
T (mutant)	9 (0.214)	2 (0.0208)		
eNOS -786T>C				
T/T	6 (28.5%)	42 (87.5%)	0.0571 (0.0159 – 0.204)	<0.0001
T/C	14 (66.6%)	6 (12.5%)	14.0 (4.02 – 48.73)	< 0.0001
C/C	1 (4.76%)	0		NS
Alleles				
T (wild)	26 (0.619)	90 (0.937)	0.1083 (0.0384 −0.305)	< 0.0001
C (mutant)	16 (0.380)	6 (0.0625)		

† by Fisher’s exact test two tailed, sample frequency expressed as no.(%), NS- not significant

**Table 2:** Genotype and allele frequencies for various eNOS polymorphisms in SCD patients with late menarche and early menarche.

Genotype or alleles	SCD late menarche (n=21)	SCD early menarche (n=18)	Odds ratio†(95% confidence interval)	*P* value†
eNOS 4a/b				
4b/4b	6 (28.5%)	17(94.4%)	0.0235 (0.0025 – 0.2188)	<0.0001
4a/4b	13 (62.0%)	1(5.5%)	27.6 (3.05 – 249.6)	0.003
4a/4a	2 (9.5%)	0		NS
Alleles				
4b (wild)	25 (0.595)	35 (0.972)	0.042 (0.00524 – 0.3369)	<0.0001
4a (mutant)	17 (0.404 )	1 (0.027)		
				
eNOS 894G>T				
G/G	13 (62.0%)	17 (94.4%)	0.095 (0.01058 – 0.8638)	0.0232
G/T	7 (33.3%)	0	19.1 ( 1.0 – 363.8)	0.0096
T/T	1 (4.7%)	1(5.5%)		NS
Alleles				
G (wild)	33 (0.785)	34 (0.944)	0.215 (0.043 – 1.07)	0.0553
T (mutant)	9 (0.214)	2 (0.055)		
				
eNOS -786T>C				
T/T	6 (28.5%)	14 (77.7%)	0.1143 (0.0265 – 0.492)	0.0036
T/C	14 (66.6%)	4 (22.2%)	7.0 (1.66 – 29.4)	0.0096
C/C	1 (4.76%)	0		NS
Alleles				
T (wild)	26 (0.619)	32 (0.888)	0.2031 (0.0604 – 0.682)	0.0089
C (mutant)	16 (0.380)	4 (0.111)		

† by Fisher’s exact test two tailed, sample frequency expressed as no.(%), NS- not significant.

**Table 3:** Distribution of eNOS gene haplotype frequencies between SCD patients with late menarche and control group cum SCD patients with early menarche.

eNOS Haplotypes †	eNOS 4a/b	eNOS 894 G>T	eNOS 786 T>C	Control + SCD early menarche	SCD Late menarche	Odds ratio (95% confidence interval)	*P* value†
Hap 1	4b	G	T	90.10%	33.30%	14.88 (5.308 – 41.68)	< 0.0001
Hap 2	4a	G	C	2.20%	21.40%	0.051 (0.0126 – 0.205)	< 0.0001
Hap 3	4b	G	C	3.70%	11.90%	0.188 (0.0504 – 0.704)	0.0179

† by Fisher’s exact test two tailed,

† Haplotype by SNP Alyzer ver 8.0

**Table 4:** Comparison of median plasma nitrite (NO2) concentration from various genotypes of eNOS polymorphisms between SCD patients with late menarche and SCD patients with early menarche combined with control individuals.

Polymorphism	Genotype	SCD early menarche + Control individuals			SCD late menarche individuals		
		Plasma Nitrite(NO_2_) conc (μM/L)	n	*P*^b^	Plasma Nitrite(NO_2_) conc (μM/L)	n	*P*^b^
eNOS 4a/b	b/b	265.6 (260.4 – 271.6)^a^	60		252.0 ( 244.6 – 278.6)^a^	6	
	a/b + a/a	266.3 (256.6 – 275.2)	6	0.937	125.3 (105.6 – 144.5)	15	0.0005
eNOS 298G>T	G/G	265.4 (260.3 – 272.3)	63		274.6 (266.0 – 284.0)	13	
	G/T + T/T	270.2( 255.0 – 277.3)	3	0.793	184.2 (155.6 – 201.8)	8	0.0002
eNOS-786T>C	T/T	265.4 (258.8 – 272.1)	56		262.9 (244.3 – 298.6)	6	
	T/C + C/C	269.3 (263.5 – 272.3)	10	0.41	142.3 (134.5 – 215.6)	15	0.0005

a The numbers in parentheses are interquartile ranges.

b *P* values obtained by Mann-Whitney *U* test.

**Table 5:** *D*′ values and *P* values for linkage disequilibrium between eNOS SNPs among the studied population.

	eNOS 4a/b	eNOS 894 G>T	eNOS - 786T>C
**eNOS 4a/b**	...	0.511	0.674
**eNOS 894 G>T**	0.0025	...	0.179
**eNOS-786T>C**	0.0137	0.961	....

*D*′ values and *P*-values analysed by SNPAlyzer ver 8.0 software platform. Above diagonal: *D*′ values in bold letter. Below the diagonal *P*-values.
